# Remotely Piloted Aircraft System (RPAS)-Based Wildlife Detection: A Review and Case Studies in Maritime Antarctica

**DOI:** 10.3390/ani10122387

**Published:** 2020-12-14

**Authors:** Chang-Uk Hyun, Mijin Park, Won Young Lee

**Affiliations:** 1 Center of Remote Sensing and GIS, Korea Polar Research Institute, Incheon 21990, Korea; chyun@kopri.re.kr; 2 Division of Life Sciences, Korea Polar Research Institute, Incheon 21990, Korea; mijin.park@kopri.re.kr; 3 School of Biological Sciences, Seoul National University, Seoul 08826, Korea

**Keywords:** wildlife biology, remotely piloted aircraft system, UAV, drone, quantitative monitoring, polar region, thermal-imaging sensor

## Abstract

**Simple Summary:**

Remotely piloted aircraft systems (RPAS) have been successfully applied in wildlife monitoring with imaging sensors to improve or to supplement conventional field observations. To effectively utilize this technique, we reviewed previous studies related to wildlife detection with RPAS. First, this study provides an overview of the applications of RPAS for wild animal studies from the perspective of individual detection and population surveys as well as behavioral studies. In terms of the RPAS payload, applying thermal-imaging sensors was determined to be advantageous in detecting homeothermic animals due to the thermal contrast with background habitat using case studies detecting southern elephant seal (*Mirounga leonina*) using RGB and thermal imaging sensors in King George Island, maritime Antarctica.

**Abstract:**

In wildlife biology, it is important to conduct efficient observations and quantitative monitoring of wild animals. Conventional wildlife monitoring mainly relies on direct field observations by the naked eyes or through binoculars, on-site image acquisition at fixed spots, and sampling or capturing under severe areal constraints. Recently, remotely piloted aircraft systems (RPAS), also called drones or unmanned aerial vehicles (UAV), were successfully applied to detect wildlife with imaging sensors, such as RGB and thermal-imaging sensors, with superior detection capabilities to those of human observation. Here, we review studies with RPAS which has been increasingly used in wildlife detection and explain how an RPAS-based high-resolution RGB image can be applied to wild animal studies from the perspective of individual detection and population surveys as well as behavioral studies. The applicability of thermal-imaging sensors was also assessed with further information extractable from image analyses. In addition, RPAS-based case studies of acquisition of high-resolution RGB images for the purpose of detecting southern elephant seals (*Mirounga leonina*) and shape property extraction using thermal-imaging sensor in King George Island, maritime Antarctica is presented as applications in an extreme environment. The case studies suggest that currently available cost-effective small-sized RPAS, which are capable of flexible operation and mounting miniaturized imaging sensors, and are easily maneuverable even from an inflatable boat, can be an effective and supportive technique for both the visual interpretation and quantitative analysis of wild animals in low-accessible extreme or maritime environments.

## 1. Introduction

In wildlife management or conservation, scientists monitor animal populations and make behavior observations from the monitoring with the quantitative information on the status and trends of populations (e.g., [1,2]). To conduct ecological monitoring on wild animals, traditional field surveys, i.e., direct field observations by the naked eyes or through binoculars, on-site image acquisition at fixed spots, and sampling or capturing under areal constraints, have been widely used. For birds, direct observation recorded by point or transect counts is typically performed within a limited time or until a cumulative richness is saturated [3]. Non-invasive samples from trapping camera or collecting feces are analyzed to apprehend the genetic relationship [4] or reproductive events [5]. Mist-netting has been mainly deployed to catch birds [6] and bats [7]. Animals captured by any other manner are banded or tagged and then released [8,9].

To observe and monitor wildlife, remote sensing techniques are considered efficient at various spatial and temporal scales [10]. Manned aircraft, a remote sensing platform, have been used to investigate the status and distribution of free-ranging wild animal populations while overcoming the aerial constraints of the conventional fieldwork-based monitoring methods (e.g., [11,12]). Aerial surveys with manned aircraft require high operating costs, especially for repetitive flights for monitoring purposes, even when investigating a small study area and with a runway within a suitable distance to the study area. This method is adequate for applications with large enough animals, such as medium to large mammals, detectable from relatively higher flight heights than remotely piloted aircraft systems (RPAS). Publicly available satellite images of regional-scale coverage from Terra and Aqua satellites with moderate-resolution imaging spectroradiometer (MODIS) or Landsat satellite data were commonly used to monitor land-cover types (e.g., [13,14]), but these mid- to low-resolution satellite images are not feasible for the direct monitoring of individual animals. Researchers indicated the problems of higher-resolution commercial satellite imagery (i.e., the spatial resolution of less than a few meters) suitable for ecological monitoring such as the high cost of scenes, limited repeat times depending on orbits, and cloud contamination and suggested to the use of lightweight unmanned aerial vehicles to solve those issues and allow researchers to study spatial ecology at close range [15].

Before describing the applications of RPAS in wildlife studies, we will clarify our use of terminology. There are several terms to indicate aerial-vehicle systems that are remotely controlled without human pilots. First, “drone” is a colloquial term to refer to a pilotless aircraft, and drones were also originally developed under military objectives [16]. As described in Table 1, “unmanned aerial vehicles (UAVs)” and “unmanned aircraft systems (UAS)” were adopted in the past [17,18]. “Remotely piloted aircraft systems (RPAS)” functioning in safe and predictable operation regarding their interaction with other aircrafts [18] are technically included in UAS; however, RPAS has now become more of an internationally adopted term compared UAS followed by the International Civil Aviation Organization (ICAO) [19] because of a widened meaning of UAS [16]. As an aircraft could be lighter or heavier than air, unmanned aircraft also includes free balloons and fully autonomous operations [19]. However, RPA operators must be properly certificated or licensed under local or international authorities with regard to aviation safety. Therefore, while the terms UAV and UAS are still widely used in many articles (e.g., [20,21]), we primarily use “RPAS” when indicating an aerial system throughout the text to highlight the importance of real-time control with RPAS.

RPAS has merits in wildlife detection and monitoring: safety [22], reasonable prices [23], and flexibility in use [24]. An RPAS could fly to an area that is dangerous for manned aircraft to approach, such as narrow fjords [25] and fragmented sea ice [26]. An RPAS could ensure the safety of researchers from becoming too close to fierce wild animals, like the Nile crocodile (*Crocodylus niloticus*) [27] and poachers [28,29]. Cost-effectiveness has long been one of the main reasons for applying RPAS as the equipment price of RPAS is at least ten times cheaper than that of a helicopter [30].

RPAS are utilized in wildlife studies for a wide range of target animals and detection purposes, so the types and functions of RPAS should be appropriately selected among their different kinds to accomplish those goals. RPAS are traditionally classified by size and wing type. For example, Anderson and Gaston [15] outlined four classes of platforms by size: large, medium, small, and mini, in addition to micro and nano. Considering the target area, larger aircraft can typically fly for longer and support a heavier payload, which is generally from the batteries.

Many RPAS designs were developed and tested for stability: fixed- and rotor-wing aircraft are popular, but experimental or biomimetic designs, with the hummingbird shape as an example, were also developed and introduced [31,32,33]. Rotor-wing aircraft can be produced with a high degree of freedom in the number of rotors, like bicopters, tricopters, or quadcopters [34]. Quadcopters, hexacopters, and octacopters are used the most due to their high reliability [34], and rotor-wing aircraft are adequate for uneven and inhomogeneous ground [35]. Rotor-wing aircraft fly slower than fixedwing aircraft can, even when hovering, and pilots may easily offhandedly change the flight path. When topological features are contemplated, the operation speed and flight path must be skillfully determined.

As research using RPAS has recently become popular, papers dealing with reviews on specific aspects of wildlife research using RPAS have been published. In view of RPAS image processing techniques, Chabot and Francis [36] provided an overview of image analysis techniques for automated bird detection and counting using manned aircraft- and RPAS-based high-resolution aerial images. Hollings et al. [37] focused on image analysis techniques for detecting and counting animals to produce animal abundance data using high-resolution images from satellite and RPAS data. Christin et al. [38] covered the use of RPAS-based images for identification and classification, behavioral studies, population monitoring, and ecosystem management and conservation using deep learning as a part of the applications in ecology.

On the other hand, Johnston [39] reviewed RPAS applications within a subdiscipline, i.e., marine science and conservation, by selecting a specific environment type. As another example of selecting an analysis target narrowed by the environment type, Verfuss et al. [40] also reviewed RPAS applications for the detection and monitoring of marine fauna. Mangewa et al. [41] analyzed previous studies applying the RPAS techniques to community wildlife management areas in Tanzania. Burke et al. [42] focused on the optimization of RPAS-based thermal imaging strategies, considering the thermal effects of ground and atmosphere, occlusion by vegetation, and effective flight height for accurate image acquisition and identification of animals of interest. Wang et al. [43] reviewed previous wild animal survey studies using satellites, manned aircraft, and RPAS and focused on the data specifications, animal detection methods, and detection accuracy.

Here, we review studies on the use of RPAS and their application on wild animal monitoring from the perspective of individual detection and population surveys as well as behavioral studies, in addition to the applicability of thermal-imaging sensors, and further extractable information from thermal image analyses. Additionally, to differentiate from existing reviews, RPAS-based case studies of the practical acquisition of high-resolution RGB images for the purpose of detecting southern elephant seal (*Mirounga leonina*) through the operation of an inflatable boat-based RPAS in Potter Peninsula, King George Island, maritime Antarctica, and the southern elephant seal detection and shape property extraction using RGB and thermal-imaging sensors in the Barton Peninsula, King George Island, maritime Antarctica are presented as applications in an extreme environment.

## 2. RPAS Application for Wild Animal Detection and Monitoring

### 2.1. Literature Review

**Figure 1:**
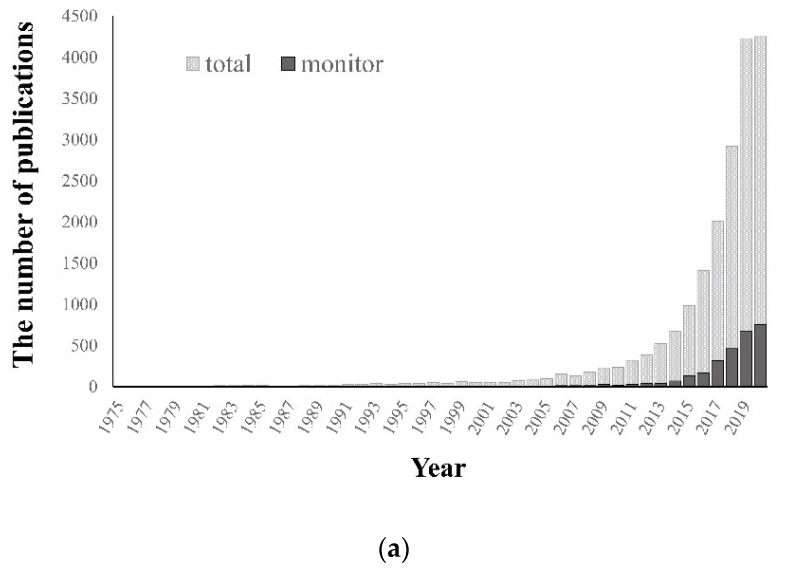

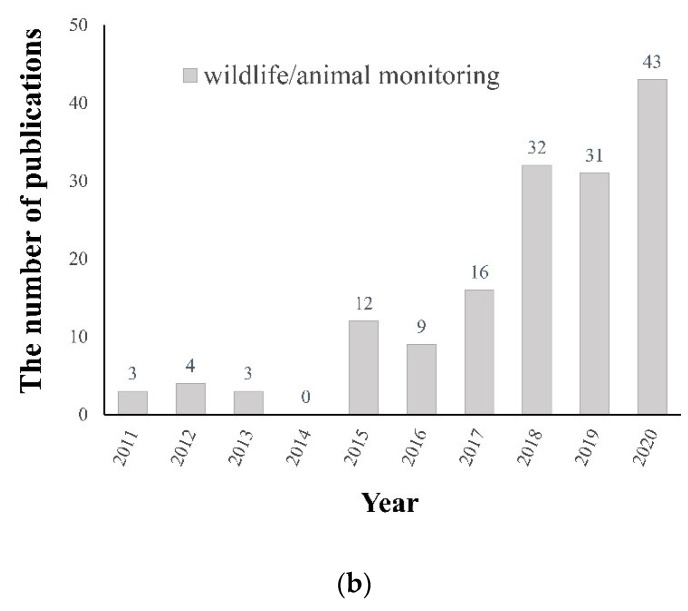
The number of publications related to remotely piloted aircraft systems (RPAS) and animal monitoring. (**a**) Web of Science search for articles containing topics “unmanned aerial” or “unmanned aircraft” or “remotely piloted aerial” or “remotely piloted aircraft” or “drone” but not “honey bee”, yielding 19,399 publications, refined with “monitoring”, obtaining 2807, from 1975 until November 2020. (**b**) The number of studies using RPAS for animal monitoring during the last ten years.

We browsed the Web of Science (http://www.webofknowledge.com) to search for articles that contained the topics of “unmanned aerial”, “unmanned aircraft”, “remotely piloted aerial”, “remotely piloted aircraft”, or “drone” but not “honey bee” in the title, abstract, and keywords. Yielding 19,399 such results from 1975 in November 2020, we chose these to wholly include the broad term UAS and formally accepted term RPAS [16]. When we included “drone”, we excluded the term with “honeybee” to reduce any confusions because drone is also used to indicate a male honeybee, *Apis mellifera*. The record of published articles to date is almost exponentially increasing and records increased by around 40% in the five most recent years (Figure 1a).

Then, we narrowed the topic down with “monitor” (*n* = 2807), which occupied 14.4% of the total articles (Figure 1a), and then “wildlife OR animal” (*n* = 157; Figure 1b and Supplementary Table S1). We primarily reviewed these searched articles and their cited references, leading to the selection of 121 articles for wild animal studies, and we categorized them according to their monitoring purposes (Supplementary Table S1). We classified wild animal monitoring applied with RPAS into individual detection and population surveys focusing on animals themselves as well as behavioral studies revealing the relationship with other animals or the surroundings. Here, we introduced selected studies for animal monitoring with RPAS into the two categories (‘individual detection and population survey’ and ‘behavioral study’) (Table 2). All studies in Figure 1b are provided in Supplementary Table S1.

**Figure 2:**
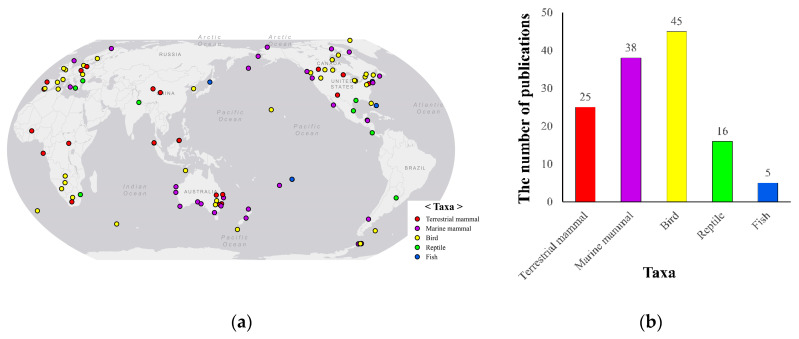
Global areas with RPAS wild animal monitoring and the number of publications. (**a**) Areas where RPAS monitoring has been applied and (**b**) the number of publications by animal taxa.

Early studies in animal monitoring tested the feasibility of RPAS with decoys [54,55] or inflatable kayaks representing whale-like targets [56], and RPAS is now applied in the field-study area. Chabot and Bird [57] argued that RPAS would fit in animal research due to nondestructive high-resolution imagery and repeatable operations, even in inaccessible areas. Therefore, RPAS is globally used regardless of latitude (Figure 2). Studies are not limited to terrestrial animals. Gharials (*Gavialis gangeticus*) and muggers (*Crocodylus palustris*) [58], dugongs (*Dugong dugon*) [59], and other aquatic animals living in freshwater and seawater have also been targeted. As depicted in Figure 2, studies on wild mammals and birds occupy a high proportion, but several attempts on reptiles [52,58,60] and fish [30,53] have been made.

### 2.2. Individual Detection and Population Survey

One of the most common research subjects in wild animal monitoring with RPAS is individual detection by human verification to observe if a target species exists in a given area. RPAS-based individual detection and population surveys extended include identifying and counting birds or mammals in cryptic habitats. When the RPAS is equipped with a thermal camera, the results can be used to easily discriminate homeothermic animal body temperatures from the surrounding backgrounds, such as rainforests with a partially exposed body through openings between leaves and clouds, preventing thermal radiation from passing through (e.g., detecting orangutans [61]) and sea ice revealing a high thermal contrast between bodies and snow/ice surface (e.g., identifying pink-footed geese (*Anser brachyrhynchus*) [44]). To improve detection quality, thermal cameras were used with RGB cameras [28,49,62,63], and white-spotted shapes were discovered in grayscale thermal images that could even detect small cryptic nests [44,51,64].

Aerial photographs from RPAS are also efficient for counting herd numbers. As animals often form groups in their natural habitats, high-resolution images are effectively acquired within an adequate operation radius of RPAS (e.g., a few kilometers) and used to estimate the population size for large animals, like hippos [45] and sharks [46]. Thus, RPAS can be applied to examine the density or distribution of cryptic or endangered species. RPAS could save time and help with identifying whole raptor nestlings and eggs [65] or Sumatran orangutan (*Pongo abelii*) nests [66], both located at the tops of trees.

As an extension of individual detection, RPAS showed strength in population surveys by saving time [67] and reconfirming field images [68]. For example, Hodgson et al. [69] put decoys on the beach so that the real number of fake wildlife could be confirmed. RPAS methods counted wildlife with less variation and in more accurate numbers than a group of human researchers by direct observation with binoculars, showing that the benefit from counting large flocks of mammals and birds using RPAS [70]. Newly designed automated image processing methods have been suggested, such as automated target detection algorithms [36,71,72] and deep-learning-based object-detection methods [47]. These attempts were all based on distinguishing the shape of the target species from the background.

Some rare cases attempted to monitor animals in a nonoptical manner, and bioacoustic detection and classification are used with pattern recognition [73] or the means to acquire samples originated from wild animals [74,75,76,77]. It can be complicated to combine the bioacoustic approach with an RPAS due to the operational noise of the aircraft. However, songbird surveys were conducted by RPAS-based bioacoustic monitoring [48], and echolocation calls of Brazilian free-tailed bats (*Tadarida brasiliensis*) were recorded [24,78]. Thus, a new method for aerial bioacoustics was demonstrated. Furthermore, an intriguing approach with RPAS to collect fresh respiratory vapor of cetacean species revealed a novel way to sample whale blow microbiome or virome [74,75,76,77]. Potential pathogens or body conditions of marine mammals could be discovered with the aid of RPAS.

### 2.3. Behavioral Study

In addition to detecting and counting certain species, recent studies have also revealed preferred habitats and behavioral observation based on the individual detection results. For example, an RPAS was utilized in analyzing changes in the ecosystem structure attributed to two introduced Eurasian beavers (*Castor fiber*) [79]. Likewise, pictures of wetlands taken at 274 m height were spatially analyzed, and least bitterns (*Ixobrychus exilis*) were shown to favor cattails for nesting [80,81]. The Global Positioning System (GPS) data loggers were attached to a couple of lesser kestrels (*Falco naumanni*), and their tracks were imitated by RPAS to understand their landscape using mosaicked images [82].

RPAS can be launched near the sea surface to observe the behavior of marine species. A case study was the recording of the courtship and mating behavior of the green turtle (*Chelonia mydas*) in the western Gulf of Mexico [52]. Another case was the application of UAVs to capture the shoaling behavior of blacktip reef sharks (*Carcharhinus melanopterus*) in Mo’orea, French Polynesia [53]. Behavioral studies using RPAS also contain video recordings or a series of images to estimate animals’ mating and courtship behavior (green turtles [52]) or collective shoaling behavior (reef sharks [53]).

Animal vocal behavior was also collected from animals. For instance, echolocating bats can be sampled by designing the vehicle with noise-cancelled flying [78]. In marine animal studies, an RPAS video survey has advantages in minimizing human-induced disturbance and reducing the cost of observing the animals compared to traditional monitoring methods using ships. In addition, new algorithms have been developed to detect swarming behavior for the aggregation and foraging of social animals [83,84]. This approach has been recently applied to collect blow samples by flying over a group of dolphins [77] and to observe the socializing and nurturing behavior of baleen whales [85]. Collectively, RPAS has been widely applied to collect multiple types of behavior by video-recordings or serial photo images as well as spatial information of animal distributions and densities.

Despite the increase of RPAS monitoring on wild animals, investigating the possible behavioral impact on individuals is scarce. Researchers have manipulated the definition of disturbance with vigilance or escape behavior [86,87,88,89]. Such responses are apparent to notice; however, this does not imply that approaching devices does not influence animals at all if there is no response. Watershed research indicated that the heart rate of American black bears (*Ursus americanus*) was elevated to about double after a UAV flew over them, while their movement rate did not show a significant difference [90]. Therefore, the aircraft must be operated on the basis of the precautionary principle, so the operator should practice beforehand, choose an appropriate aircraft and sensor, and report flight details such as altitude, speed, and angle [91,92].

## 3. RPAS Use in Extreme Environments: Applications on Arctic and Antarctic Research

One of the major advantages of deploying RPAS is to secure safety. Cautiously operated RPAS reduces unexpected damage to human life by not piloting a manned aircraft or not exploring an unfamiliar area firsthand. This advantage produces fruitful results in extreme environments such as the polar regions [26,44] and deserts [93] or topographically inaccessible zones that researchers cannot easily approach, such as seaside cliffs [94].

The Arctic and Antarctic regions are difficult regions for monitoring research due to their cold and windy environments. RPAS are preferred when exploring inaccessible areas like oceans with floating ice or fjords. Ribbons (*Histriophoca fasciata*) and spotted seals (*Phoca largha*), referred to as ice-associated seals, were monitored by a fixed-wing aircraft at the Bering Sea ice edge [26]. In addition, sudden gusts of wind can occur in narrow fjords, which is risky for piloting manned aircraft; hence, a UAV was designated for a transect survey of the humpback whale (*Megaptera novaeangliae*) and killer whale (*Orcinus orca*) [25]. Disturbances on these marine animals are easily overlooked because they appear to have no need to defend against aerial predators due to their huge size. However, bottlenose dolphins (*Tursiops truncatus*) and Antillean manatees (*Trichechus manatus manatus*) were found to respond to small quadcopters even at altitudes of 30 and 104 m [88].

Penguins, as colonial birds, nest close to each other in open space and this makes it difficult for researchers to estimate their population. Ratcliffe et al. [95] operated a UAV to survey a gentoo penguin (*Pygoscelis papua*) colony at Volunteer Point, East Falkland in sub-Antarctic islands. At 30 m height above ground, the images of penguins clearly revealed individuals, and 1021 active nests were found in the mosaicked images covering the whole colony. Similarly, Goebel et al. [96] took aerial photographs of the gentoo and chinstrap penguin (*Pygoscelis antarctica*), Antarctic fur seals (*Arctocephalus gazelle*), Weddell seals (*Leptonychotes weddellii*), and leopard seals (*Hydrurga leptonyx*) at Cape Shirreff, Livingston Island, South Shetland Islands.

In the polar regions, thermal sensors are effective because the surface temperature of ice or seawater is contrasted from that of endothermic animals. Thermal images were applied onto two grey seal (*Halichoerus grypus*) colonies to develop automated detection and enumeration [72].

Here, we present an RPAS-based case study of the practical acquisition of high-resolution RGB images for the purpose of investigating southern elephant seal through the operation of an inflatable boat-based RPAS in Potter Peninsula, King George Island, Antarctica. In Antarctica, berthing facilities are rare; therefore, takeoff and landing on board can be a feasible method of RPAS operation for investigating coastal animals.

**Figure 3:**
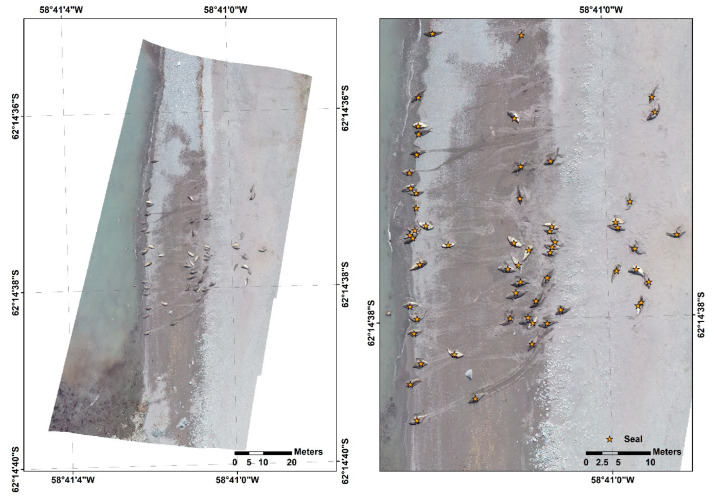
Mosaicked RPAS-based RGB images for identifying the status of Southern elephant seals (*Mirounga leonina*) in Potter Peninsula, King George Island, Antarctica.

In December 2019, Phantom 4 (DJI Co., Shenzhen, China) was controlled from an inflatable boat anchored near Potter Peninsula and acquired four images over the coast. The images were mosaicked using Metashape 1.6.2 software (Agisoft LLC, St. Petersburg, Russia) with a spatial resolution of 3.0 cm (Figure 3). In the mosaicked image, a total of 60 individuals were detected by human verification. From this case study, the Phantom 4, a small-sized RPAS, can be evaluated as a practical RPAS from its easy takeoff and landing function even on a small boat for high-resolution RGB image acquisition, aiming coast animal monitoring in maritime Antarctica.

## 4. RPAS-Based Thermography in Wild Animal Monitoring

Thermal cameras can be used together with high-resolution RGB cameras [28,62,63] to complement the lower spatial resolution of a thermal detector, e.g., 640 × 512 pixels [44,50,64]. Although small-scale surface features are omitted in thermal images due to the larger pixel size, the thermal contrast between wild animals and the background land covers enables identifying individuals or aggregations [72]. The thermal contrast yielded from differences in the surface temperature vary from the composition of land cover with a combination of environmental factors, such as the duration of exposure to the sun and shadow casting from the local topography. Therefore, for optimal monitoring, the timely acquisition of a thermal image preventing a homogenized scene temperature is crucial to maximize the detectability of target animals by enhancing the thermal contrast between targets and backgrounds [42,97].

### 4.1. Detection and Counting Wild Animals Using Thermal Images

RPAS-based thermography was used to automatically detect marine wildlife like the grey seal (*Halichoerus grypus*), with manually selected temperature and polygon area thresholding to develop automated detection and enumeration [72]. Although the intrinsically low resolution of the thermal image is an obstacle to clearly depict individual targets, pixel aggregations above the temperature threshold were able to be separated to individual seals using high pass-filtering to estimate the population of seals. Particularly, in the Arctic and Antarctic regions, the detection capability of endothermic animals can be emphasized from the higher thermal contrast between targets and backgrounds [44].

### 4.2. Retrieving Quantitative Properties from RPAS-Based Thermal Images

Quantitative properties, including the length and mass of individual animals, have been retrieved from high-resolution RGB images. Comparing with traditional measurement protocols [98], RPAS-based methods showed a higher accuracy to predict seal mass with the photogrammetric standard length of leopard seals [99]. Lee et al. [44] identified the approximate size of pink-footed geese by calculating the longest length of the convex hull of manually selected isothermal polygons depicting individual birds.

**Figure 4:**
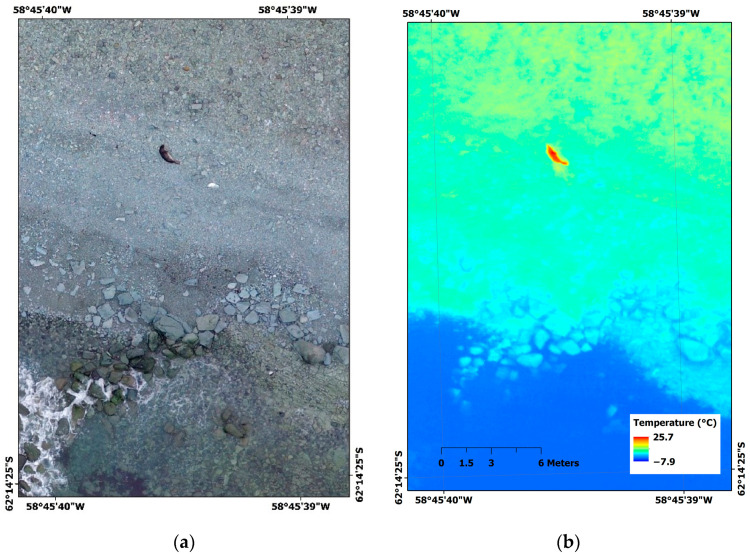
Southern elephant seal (*Mirounga leonina*) captured by RPAS-based RGB and thermal images in Barton Peninsula, King George Island, Antarctica. (**a**) Mosaicked RGB image and (**b**) coregistered mosaicked thermal image to mosaicked RGB image.

Here, we present an example of retrieving a quantitative feature, the curvilinear overall length [99] of the southern elephant seal, using RPAS-based thermal images acquired in the Barton Peninsula, King George Island, Antarctica. A DJI Inspire V1 with a 12-megapixel Zenmuse X3 RGB camera (DJI Co., Shenzhen, China) and FLIR Vue Pro R thermal-imaging sensor with 13 mm lens (FLIR Systems, Wilsonville, OR, USA) were operated on 4 January 2020 with Pix4D Capture flight planning mobile application (Pix4D S.A., Lausanne, Switzerland). Individual raw radiometric JPEG files from the thermal-imaging sensor were converted to Geotiff format files using FLIR ResearchIR software (FLIR Systems, Wilsonville, OR, USA) for further processing. RGB images and thermal images were mosaicked using Metashape 1.6.2 software (Agisoft LLC, St. Petersburg, Russia) with a spatial resolution of 1.8 and 5.7 cm, respectively; then, the mosaicked thermal image was coregistered to the mosaicked RGB image of finer spatial resolution and higher geolocation accuracy using a georeferencing tool in ArcGIS 10.3 software (ESRI, Redlands, CA, USA) (Figure 4). The seal was clearly identifiable in the thermal image from the background of seashore gravel.

**Figure 5:**
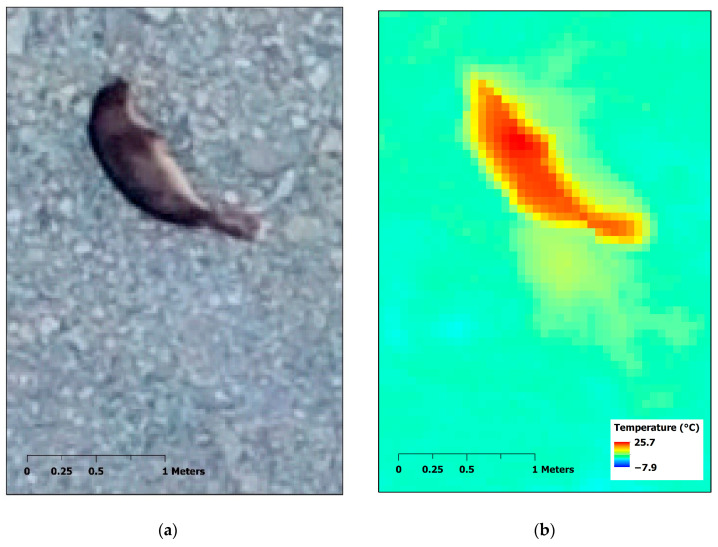

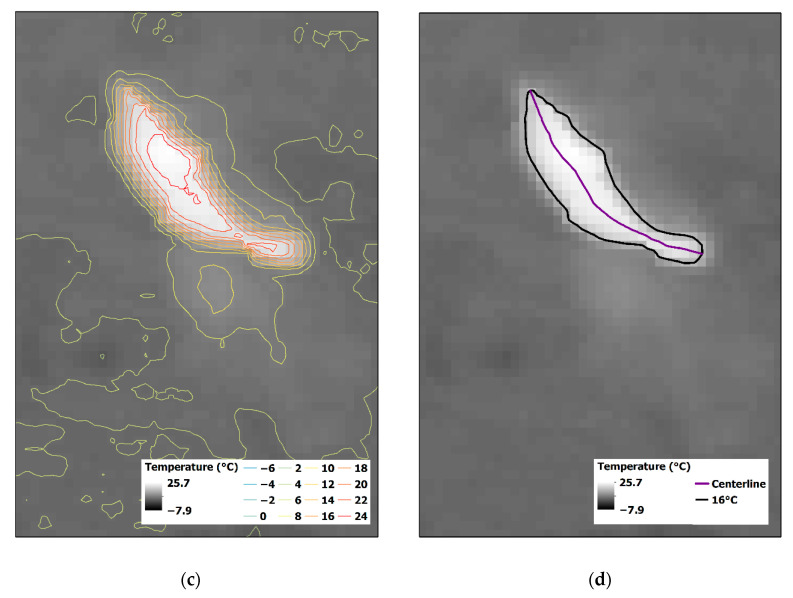
Mosaicked RPAS-based RGB and thermal images zoomed to a Southern elephant seal (*Mirounga leonina*): (**a**) zoomed RGB image, (**b**) zoomed coregistered thermal image, (**c**) temperature contours with 1°C interval overlaid on thermal image, and (**d**) selected contour of 16 °C depicting the highest thermal contrast and centerline of the polygon indicating an individual seal.

The shape of the seal was recognizable in the thermal image, similar to the higher-resolution RGB image (Figure 5a,b). To retrieve the seal size, temperature contours with 1 °C intervals were delineated as polygons using ArcGIS (Figure 5c). The smallest change in the area of polygons between adjacent temperatures indicating the highest thermal contrast was considered as the threshold temperature, i.e., 16 °C, for determining the boundary of the seal body (Figure 5d). To measure the overall curvilinear length, the centerline of the selected 16 °C temperature polygon was extracted using the Thiessen polygon method [100]. Then, the endpoints of the centerline were extended to vertices at the ends of the polygon to fill gaps. From the extracted centerline, the overall curvilinear length of the target seal was calculated as 170.1 cm, similar to the ca. 180 cm measured in the field using a tape measure.

Here, we examine that the RPAS-based thermal approach was efficient to detect Antarctic seals on the coastal regions and count the number of individuals. Based on the size measurements, we expect that it can be applied to distinguish small-sized sub-adults among adults. The captured seal in Figure 4 was estimated to be a sub-adult individual which was resting near the shore. When it is observed by humans with binoculars, it is often confused rocks or wet sands due to their dark-colored body. Thus, thermal images can be used to detect such small seals solely distributed. In addition, adult individuals can be further discriminated between the sexes. Since southern elephant seals have high levels of sexual dimorphisms in body sizes, a dominant alpha male can be detected from the size measurements using the RPAS images.

## 5. Conclusions

Our review showed that RPAS and imaging sensors can contribute to wild animal studies, including the identification of individuals from backgrounds, counting detected individuals, and monitoring. In addition, multi-temporal high-resolution image acquisition capability within an operation radius enables behavioral studies. Geographically registered RPAS-based images can also be used to measure animal properties, e.g., body size. Therefore, RPAS is a useful technique to detect and monitor wild animals, particularly in extreme environments such as polar regions, by helping researchers find cryptic animals and nests in more secured fieldwork conditions. Although an RPAS can offer an effective and efficient method for animal detection, it still cannot totally replace the current monitoring methods of conventional surveys by researchers themselves. RPAS-based images may miss actual animal figures depending on the light conditions and environmental obstacles, e.g., clouds, leaves, and topographic hindrance. Yet, we suggest using an RPAS for collecting on-site material to support survey data for various purposes and expect using RPAS in wildlife studies will be expanded continuously according to its potential uses and RPAS and sensor technology advances.

## Figures and Tables

**Table 1:** Term glossaries indicating aerial-vehicle systems that are remotely controlled without human pilots.

Acronym	Definition
Unmanned aerial vehicle (UAV)	A pilotless aircraft that is flown without a pilot-in-command on-board is either remotely and fully controlled from another place (ground, another aircraft, or space) or programmed and fully autonomous [17]
Unmanned aircraft system (UAS)	An aircraft and its associated elements that are operated with no pilot on board [18]
Remotely piloted aircraft (RPA)	An unmanned aircraft that is piloted from a remote pilot station [19]
Remotely piloted aircraft system (RPAS)	A remotely piloted aircraft, its associated remote pilot station(s), the required command and control links, and any other component as specified in the type design [19]

**Table 2:** Selected applications of RPAS on wild animal monitoring.

Subject	Species	Area	Sensor Used	Studies of Importance	Reference
Individual detection and population survey	Pink-footed goose (*Anser branchyrhynchus*), common ringed plover (*Charadrius hiaticula*), black-faced spoonbill (*Platalea minor*)	Sirius Passet, Greenland and Yellow Sea, Republic of Korea	Phantom 4 (DJI Co., Shenzhen, China) and a thermal camera (FLIR Systems, Wilsonville, OR, USA)	Detected three bird species in inaccessible areas	[44]
	Common hippopotamus (*Hippopotamus amphibious*)	Garamba National Park, Democratic Republic of Congo	Sony Nex7 digital still camera (Sony, Tokyo, Japan)	Determined optimal flight parameters and estimated total number	[45]
	Sharks (lemon *Negaprion brevirostris*, nurse *Ginglymostoma cirratum*, bonnethead *Sphyrna tiburo*), stingrays (*Dasyatis americana*), and spotted eagle rays (*Aetobatus narinari*)	Great Abaco Island, The Bahamas	Phantom Vision 2+^®^ (DJI Co., Shenzhen, China)	Identified and counted marine megafauna in shallow habitats	[46]
	Spot-billed duck (*Anas poecilorhyncha*), greenwinged teal (*Anas crecca*), great egret (*Ardea alba*), gray heron (*Ardea cinerea*)	Shihwa lake and Yeongjong island, Republic of Korea	NX500 camera (Samsung Corp., Seoul, Republic of Korea), and Phantom 4 Pro (DJI Co., Shenzhen, China)	Compared different bird-detection models with field study and decoy	[47]
	Wood thrush (*Hylocichla mustelina*), gray catbird (*Dumetella carolinensis*), song sparrow (*Melospiza melodia*), and other songbirds	State Game Lands 249, Pennsylvania, USA	H1 Handy Recorder (Zoom Corp., Tokyo, Japan)	Developed bioacoustics and RPAS combined monitoring protocol for counting songbirds	[48]
	Wild boar (*Sus scrofa*), red deer (*Cervus elaphus*), and fallow deer (*Dama dama*)	Doñana National Park, SW Spain	Lumix LX-3 digital photo camera 11MP (Panasonic, Osaka, Japan)	Developed new spatially explicit aggregation index and applied on a field study	[49]
	Roe deer (*Capreolus capreolus*)	Lány, Czech Republic	WIRIS second-generation thermalimaging camera (Workswell, Prague, Czech Republic)	Searched for roe-deer fawns before harvesting to reduce unexpected kills	[50]
	Field sparrow (*Spizella pusilla*)	Pierce Cedar Creek Institute, Michigan, USA	Zenmuse XT thermal camera (FLIR Systems, Wilsonville, OR, USA)	Detected cryptic nests in grassland and showed the efficiency of RPAS-assisted searches	[51]
Behavioral study	Green turtle (*Chelonia mydas*)	Rancho Nuevo, Tamaulipas, Mexico	Inspire 1™ (DJI Co., Shenzhen, China)	Recorded courtship and mating behavior	[52]
	Reef shark (*Carcharhinus melanopterus*)	Society Archipelago, French Polynesia	Hero 3+ Silver edition (GoPro, San Mateo, CA, USA)	Quantified collective shoaling behavior through fixed images from video	[53]

## Supplementary Materials

The following are available online at https://www.mdpi.com/2076-2615/10/12/2387/s1, Table S1: Article information for wild animal monitoring with RPAS which had been searched on Web of Science (n = 157) and selected articles (n = 121) with detailed information on study species, sites, and purposes.

## References

[1] Morrison M.L. (1994). Resource inventory and monitoring: Concepts and applications for ecological restoration. Restor. Manag. Notes.

[2] Griffith J.A. (1997). Connecting ecological monitoring and ecological indicators: A review of the literature. J. Environ. Syst..

[3] Watson D.M. (2003). The ‘standardized search’: An improved way to conduct bird surveys. Austral. Ecol..

[4] Galaverni M., Palumbo D., Fabbri E., Caniglia R., Greco C., Randi E. (2012). Monitoring wolves (*Canis lupus*) by non-invasive genetics and camera trapping: A small-scale pilot study. Eur. J. Wildl. Res..

[5] Graham L.H. (2004). Non-invasive monitoring of reproduction in zoo and wildlife species. Annu. Rev. Biomed. Sci..

[6] Silkey M., Nur N., Geupel G.R. (1999). The use of mist-net capture rates to monitor annual variation in abundance: A validation study. Condor.

[7] MacCarthy K.A., Carter T.C., Steffen B.J., Feldhamer G.A. (2006). Efficacy of the mist-net protocol for Indiana bats: A video analysis. Northeast. Nat..

[8] Stamm D.D., Davis D.E., Robbins C.S. (1960). A method of studying wild bird populations by mist-netting and banding. Bird-Band..

[9] Thorstrom R., Watson R., Damary B., Toto F., Baba M., Baba V. (1995). Repeated sightings and first capture of a live Madagascar Serpent-eagle Eutriorchis astur. Bull. Br. Ornithol. Club.

[10] Willis K.S. (2015). Remote sensing change detection for ecological monitoring in United States protected areas. Biol. Conserv..

[11] Reading R.P., Mix H.M., Lhagvasuren B., Feh C., Kane D.P., Dulamtseren S., Enkhbold S. (2001). Status and distribution of khulan (Equus hemionus) in Mongolia. J. Zool..

[12] Nellemann C., Vistnes I., Jordhøy P., Strand O. (2001). Winter distribution of wild reindeer in relation to power lines, roads and resorts. Biol. Conserv..

[13] Friedl M.A., McIver D.K., Hodges J.C., Zhang X.Y., Muchoney D., Strahler A.H., Woodcock C.E., Gopal S., Schneider A., Cooper A. (2002). Global land cover mapping from MODIS: Algorithms and early results. Remote Sens. Environ..

[14] Cohen W.B., Goward S.N. (2004). Landsat’s role in ecological applications of remote sensing. Bioscience.

[15] Anderson K., Gaston K.J. (2013). Lightweight unmanned aerial vehicles will revolutionize spatial ecology. Front. Ecol. Environ..

[16] Granshaw S.I. (2018). RPV, UAV, UAS, RPAS… or just drone?. Photogramm. Rec..

[17] ICAO (2005). Global Air Traffic Management Operational Concept.

[18] ICAO (2011). Unmanned Aircraft Systems (UAS).

[19] ICAO (2015). Manual on Remotely Piloted Aircraft Systems (RPAS).

[20] Rees A.F., Avens L., Ballorain K., Bevan E., Broderick A.C., Carthy R.R., Christianen M.J., Duclos G., Heithaus M.R., Johnston D.W. (2018). The potential of unmanned aerial systems for sea turtle research and conservation: A review and future directions. Endanger. Species Res..

[21] Korczak-Abshire M., Zmarz A., Rodzewicz M., Kycko M., Karsznia I., Chwedorzewska K. (2019). Study of fauna population changes on Penguin Island and Turret Point Oasis (King George Island, Antarctica) using an unmanned aerial vehicle. Polar Biol..

[22] Christie K.S., Gilbert S.L., Brown C.L., Hatfield M., Hanson L. (2016). Unmanned aircraft systems in wildlife research: Current and future applications of a transformative technology. Front. Ecol. Environ..

[23] Weissensteiner M.H., Poelstra J.W., Wolf J.B.W. (2015). Low-budget ready-to-fly unmanned aerial vehicles: An effective tool for evaluating the nesting status of canopy-breeding bird species. J. Avian Biol..

[24] Fu Y., Kinniry M., Kloepper L.N. (2018). The Chirocopter: A UAV for recording sound and video of bats at altitude. Methods Ecol. Evol..

[25] Aniceto A.S., Biuw M., Lindstrøm U., Solbø S.A., Broms F., Carroll J. (2018). Monitoring marine mammals using unmanned aerial vehicles: Quantifying detection certainty. Ecosphere.

[26] Moreland E.E., Cameron M.F., Angliss R.P., Boveng P.L. (2015). Evaluation of a ship-based unoccupied aircraft system (UAS) for surveys of spotted and ribbon seals in the Bering Sea pack ice. J. Unmanned Veh. Syst..

[27] Ezat M.A., Fritsch C.J., Downs C.T. (2018). Use of an unmanned aerial vehicle (drone) to survey Nile crocodile populations: A case study at Lake Nyamithi, Ndumo game reserve, South Africa. Biol. Conserv..

[28] Mulero-Pázmány M., Stolper R., van Essen L.D., Negro J.J., Sassen T. (2014). Remotely piloted aircraft systems as a rhinoceros anti-poaching tool in Africa. PLoS ONE.

[29] Rey N., Volpi M., Joost S., Tuia D. (2017). Detecting animals in African Savanna with UAVs and the crowds. Remote Sens. Env..

[30] Kudo H., Koshino Y., Eto A., Ichimura M., Kaeriyama M. (2012). Cost-effective accurate estimates of adult chum salmon, *Oncorhynchus keta*, abundance in a Japanese river using a radio-controlled helicopter. Fish. Res..

[31] Mackenzie D. (2012). A Flapping of Wings. Science.

[32] Mackenzie D. (2012). It’s a Bird, It’s a Plane, It’s a… Spy?. Science.

[33] Hassanalian M., Abdelkefi A. (2017). Classifications, applications, and design challenges of drones: A review. Prog. Aerosp. Sci..

[34] Agrawal K., Shrivastav P. (2015). Multi-rotors: A revolution in unmanned aerial vehicle. Int. J. Sci. Res..

[35] Sarkisov Y.S., Yashin G.A., Tsykunov E.V., Tsetserukou D. (2018). Dronegear: A novel robotic landing gear with embedded optical torque sensors for safe multicopter landing on an uneven surface. IEEE Robot. Autom. Lett..

[36] Chabot D., Francis C.M. (2016). Computer-automated bird detection and counts in high-resolution aerial images: A review. J. Field Ornithol..

[37] Hollings T., Burgman M., van Andel M., Gilbert M., Robinson T., Robinson A. (2018). How do you find the green sheep? A critical review of the use of remotely sensed imagery to detect and count animals. Methods Ecol. Evol..

[38] Christin S., Hervet É., Lecomte N. (2019). Applications for deep learning in ecology. Methods Ecol. Evol..

[39] Johnston D.W. (2019). Unoccupied aircraft systems in marine science and conservation. Annu. Rev. Mar. Sci..

[40] Verfuss U.K., Aniceto A.S., Harris D.V., Gillespie D., Fielding S., Jiménez G., Johnston P., Sinclair R.R., Sivertsen A., Solbø S.A. (2019). A review of unmanned vehicles for the detection and monitoring of marine fauna. Mar. Pollut. Bull..

[41] Mangewa L.J., Ndakidemi P.A., Munishi L.K. (2019). Integrating UAV Technology in an Ecological Monitoring System for Community Wildlife Management Areas in Tanzania. Sustainability.

[42] Burke C., Rashman M., Wich S., Symons A., Theron C., Longmore S. (2019). Optimizing observing strategies for monitoring animals using drone-mounted thermal infrared cameras. Int. J. Remote Sens..

[43] Wang D., Shao Q., Yue H. (2019). Surveying wild animals from satellites, manned aircraft and unmanned aerial systems (UASs): A review. Remote Sens..

[44] Lee W.Y., Park M., Hyun C.-U. (2019). Detection of two Arctic birds in Greenland and an endangered bird in Korea using RGB and thermal cameras with an unmanned aerial vehicle (UAV). PLoS ONE.

[45] Linchant J., Lhoest S., Quevauvillers S., Lejeune P., Vermeulen C., Ngabinzeke J.S., Belanganayi B.L., Delvingt W., Bouché P. (2018). UAS imagery reveals new survey opportunities for counting hippos. PLoS ONE.

[46] Hensel E., Wenclawski S., Layman C. (2018). Using a small, consumer grade drone to identify and count marine megafauna in shallow habitats. Lat. Am. J. Aquat. Res..

[47] Hong S.-J., Han Y., Kim S.-Y., Lee A.-Y., Kim G. (2019). Application of deep-learning methods to bird detection using unmanned aerial vehicle imagery. Sensors.

[48] Wilson A.M., Barr J., Zagorski M. (2017). The feasibility of counting songbirds using unmanned aerial vehicles. Auk Ornithol. Adv..

[49] Laguna E., Barasona J.A., Triguero-Ocaña R., Mulero-Pázmány M., Negro J.J., Vicente J., Acevedo P. (2018). The relevance of host overcrowding in wildlife epidemiology: A new spatially explicit aggregation index. J. Ecol. Indic..

[50] Cukor J., Bartoška J., Rohla J., Sova J., Machálek A. (2019). Use of aerial thermography to reduce mortality of roe deer fawns before harvest. PeerJ.

[51] Scholten C., Kamphuis A., Vredevoogd K., Lee-Strydhorst K., Atma J., Shea C., Lamberg O., Proppe D. (2019). Real-time thermal imagery from an unmanned aerial vehicle can locate ground nests of a grassland songbird at rates similar to traditional methods. J. Biol. Conserv..

[52] Bevan E., Wibbels T., Navarro E., Rosas M., Najera B.M.Z., Sarti L., Illescas F., Montano J., Pena L.J., Burchfield P. (2016). Using unmanned aerial vehicle (UAV) technology for locating, identifying, and monitoring courtship and mating behavior in the Green Turtle (*Chelonia mydas*). Herpetol. Rev..

[53] Rieucau G., Kiszka J.J., Castillo J.C., Mourier J., Boswell K.M., Heithaus M.R. (2018). Using unmanned aerial vehicle (UAV) surveys and image analysis in the study of large surface-associated marine species: A case study on reef sharks *Carcharhinus melanopterus* shoaling behaviour. J. Fish. Biol.

[54] Abd-Elrahman A., Pearlstine L., Percival F. (2005). Development of pattern recognition algorithm for automatic bird detection from unmanned aerial vehicle imagery. Surv. Land Inf. Sci..

[55] Jones G.P., Pearlstine L.G., Percival H.F. (2006). An assessment of small unmanned aerial vehicles for wildlife research. Wildl. Soc. Bull..

[56] Koski W.R., Allen T., Ireland D., Buck G., Smith P.R., Macrender A.M., Halick M.A., Rushing C., Sliwa D.J., McDonald T.L. (2009). Evaluation of an unmanned airborne system for monitoring marine mammals. Aquat. Mamm..

[57] Chabot D., Bird D.M. (2015). Wildlife research and management methods in the 21st century: Where do unmanned aircraft fit in?. J. Unmanned Veh. Syst..

[58] Thapa G.J., Thapa K., Thapa R., Jnawali S.R., Wich S.A., Poudyal L.P., Karki S. (2018). Counting crocodiles from the sky: Monitoring the critically endangered gharial (*Gavialis gangeticus*) population with an unmanned aerial vehicle (UAV). J. Unmanned Veh. Syst..

[59] Amanda H., Natalie K., David P. (2013). Unmanned aerial vehicles (UAVs) for surveying marine fauna: A dugong case study. PLoS ONE.

[60] Biserkov V.Y., Lukanov S.P. (2017). Unmanned aerial vehicles (UAVs) for surveying freshwater turtle populations: Methodology adjustment. Acta Zool. Bulg. Suppl..

[61] Burke C., Rashman M.F., Longmore S.N., McAree O., Glover-Kapfer P., Ancrenaz M., Wich S.A. (2019). Successful observation of orangutans in the wild with thermal-equipped drones. J. Unmanned Veh. Syst..

[62] Chrétien L.P., Théau J., Ménard P. (2016). Visible and thermal infrared remote sensing for the detection of whitetailed deer using an unmanned aerial system. Wildl. Soc. Bull..

[63] Witczuk J., Pagacz S., Zmarz A., Cypel M. (2018). Exploring the feasibility of unmanned aerial vehicles and thermal imaging for ungulate surveys in forests—Preliminary results. Int. J. Remote Sens..

[64] Israel M., Reinhard A. Detecting nests of lapwing birds with the aid of a small unmanned aerial vehicle with thermal camera. Proceedings of the 2017 International Conference on Unmanned Aircraft Systems (ICUAS).

[65] Junda J., Greene E., Bird D.M. (2015). Proper flight technique for using a small rotary-winged drone aircraft to safely, quickly, and accurately survey raptor nests. J. Unmanned Veh. Syst..

[66] Wich S., Dellatore D., Houghton M., Ardi R., Koh L.P. (2016). A preliminary assessment of using conservation drones for Sumatran orang-utan (*Pongo abelii*) distribution and density. J. Unmanned Veh. Syst..

[67] McClelland G.T., Bond A.L., Sardana A., Glass T. (2016). Rapid population estimate of a surface-nesting seabird on a remote island using a low-cost unmanned aerial vehicle. Mar. Ornithol..

[68] Hurford C. (2017). Improving the accuracy of bird counts using manual and automated counts in ImageJ: An open-source image processing program. The Roles of Remote Sensing in Nature Conservation.

[69] Hodgson J.C., Mott R., Baylis S.M., Pham T.T., Wotherspoon S., Kilpatrick A.D., Raja Segaran R., Reid I., Terauds A., Koh L.P. (2018). Drones count wildlife more accurately and precisely than humans. Methods Ecol. Evol..

[70] Chabot D., Craik S., Bird D.M. (2015). Population census of a large Common Tern colony with a small unmanned aircraft. PLoS ONE.

[71] Christiansen P., Steen K.A., Jørgensen R.N., Karstoft H. (2014). Automated detection and recognition of wildlife using thermal cameras. Sensors.

[72] Seymour A.C., Dale J., Hammill M., Halpin P.N., Johnston D.W. (2017). Automated detection and enumeration of marine wildlife using unmanned aircraft systems (UAS) and thermal imagery. Sci. Rep..

[73] Frommolt K.-H., Tauchert K.-H. (2014). Applying bioacoustic methods for long-term monitoring of a nocturnal wetland bird. Ecol. Inform..

[74] Pirotta V., Smith A., Ostrowski M., Russell D., Jonsen I.D., Grech A., Harcourt R. (2017). An economical custom-built drone for assessing whale health. Front. Mar. Sci..

[75] Apprill A., Miller C.A., Moore M.J., Durban J.W., Fearnbach H., Barrett-Lennard L.G. (2017). Extensive core microbiome in drone-captured whale blow supports a framework for health monitoring. MSystems.

[76] Geoghegan J.L., Pirotta V., Harvey E., Smith A., Buchmann J.P., Ostrowski M., Eden J.-S., Harcourt R., Holmes E.C. (2018). Virological sampling of inaccessible wildlife with drones. Viruses.

[77] Centelleghe C., Carraro L., Gonzalvo J., Rosso M., Esposti E., Gili C., Bonato M., Pedrotti D., Cardazzo B., Povinelli M. (2020). The use of Unmanned Aerial Vehicles (UAVs) to sample the blow microbiome of small cetaceans. PLoS ONE.

[78] Kloepper L.N., Kinniry M. (2018). Recording animal vocalizations from a UAV: Bat echolocation during roost reentry. Sci. Rep..

[79] Puttock A.K., Cunliffe A.M., Anderson K., Brazier R.E. (2015). Aerial photography collected with a multirotor drone reveals impact of Eurasian beaver reintroduction on ecosystem structure. J. Unmanned Veh. Syst..

[80] Chabot D., Bird D.M. (2013). Small unmanned aircraft: Precise and convenient new tools for surveying wetlands. J. Unmanned Veh. Syst..

[81] Chabot D., Carignan V., Bird D. (2014). Measuring habitat quality for Least Bitterns in a created wetland with use of a small unmanned aircraft. Wetlands.

[82] Rodríguez A., Negro J.J., Mulero M., Rodríguez C., Hernández-Pliego J., Bustamante J. (2012). The eye in the sky: Combined use of unmanned aerial systems and GPS data loggers for ecological research and conservation of small birds. PLoS ONE.

[83] Bandala A.A., Dadios E.P., Vicerra R.R.P., Lim L.A.G. (2014). Swarming algorithm for unmanned aerial vehicle (uav) quadrotors–swarm behavior for aggregation, foraging, formation, and tracking. J. Adv. Comput. Intell. Intell. Inform..

[84] Vicerra R.R.P., Barcos R.N.R., Bulan J.K.S., Loteriña A.J.O., Oliver S., Pineda J.M.D., Cruz A.R.D., Roxas E.A., Bandala A.A., Dadios E.P. A comparative study of swarm foraging behaviors; trophallaxis, task allocation and pheromone. Proceedings of the 2015 International Conference on Humanoid, Nanotechnology, Information Technology, Communication and Control, Environment and Management (HNICEM).

[85] Fiori L., Martinez E., Orams M.B., Bollard B. (2020). Using Unmanned Aerial Vehicles (UAVs) to assess humpback whale behavioral responses to swim-with interactions in Vava’u, Kingdom of Tonga. J. Sustain. Tour..

[86] McEvoy J.F., Hall G.P., McDonald P.G. (2016). Evaluation of unmanned aerial vehicle shape, flight path and camera type for waterfowl surveys: Disturbance effects and species recognition. PeerJ.

[87] Rümmler M.-C., Mustafa O., Maercker J., Peter H.-U., Esefeld J. (2016). Measuring the influence of unmanned aerial vehicles on Adélie penguins. Polar Biol..

[88] Ramos E.A., Maloney B., Magnasco M.O., Reiss D. (2018). Bottlenose dolphins and antillean manatees respond to small multi-rotor unmanned aerial systems. Front. Mar. Sci..

[89] Rümmler M.-C., Mustafa O., Maercker J., Peter H.-U., Esefeld J. (2018). Sensitivity of Adélie and Gentoo penguins to various flight activities of a micro UAV. Polar Biol..

[90] Ditmer M.A., Vincent J.B., Werden L.K., Tanner J.C., Laske T.G., Iaizzo P.A., Garshelis D.L., Fieberg J.R. (2015). Bears show a physiological but limited behavioral response to unmanned aerial vehicles. Curr. Biol..

[91] Vas E., Lescroël A., Duriez O., Boguszewski G., Grémillet D. (2015). Approaching birds with drones: First experiments and ethical guidelines. Biol. Lett..

[92] Hodgson J.C., Koh L.P. (2016). Best practice for minimising unmanned aerial vehicle disturbance to wildlife in biological field research. Curr. Biol..

[93] Su X., Dong S., Liu S., Cracknell A.P., Zhang Y., Wang X., Liu G. (2018). Using an unmanned aerial vehicle (UAV) to study wild yak in the highest desert in the world. Int J. Remote Sens.

[94] Brisson-Curadeau É., Bird D., Burke C., Fifield D.A., Pace P., Sherley R.B., Elliott K.H. (2017). Seabird species vary in behavioural response to drone census. Sci. Rep..

[95] Ratcliffe N., Guihen D., Robst J., Crofts S., Stanworth A., Enderlein P. (2015). A protocol for the aerial survey of penguin colonies using UAVs (unmanned aerial vehicles). J. Unmanned Veh. Syst..

[96] Goebel M., Perryman W., Hinke J., Krause D., Hann N., Gardner S., LeRoi D. (2015). A small unmanned aerial system for estimating abundance and size of Antarctic predators. Polar Biol..

[97] He G., Yang H., Pan R., Sun Y., Zheng P., Wang J., Jin X., Zhang J., Li B., Guo S. (2020). Using unmanned aerial vehicles with thermal-image acquisition cameras for animal surveys: A case study on the Sichuan snub-nosed monkey in the Qinling Mountains. Integr. Zool..

[98] Scheffer V. (1967). Standard measurements of seals. J. Mammal..

[99] Krause D.J., Hinke J.T., Perryman W.L., Goebel M.E., LeRoi D.J. (2017). An accurate and adaptable photogrammetric approach for estimating the mass and body condition of pinnipeds using an unmanned aerial system. PLoS ONE.

[100] Dilts T. (2015). Polygon to Centerline Tool for ArcGIS.

